# Alexithymia characteristics are associated with salience network activity in healthy participants: an arterial spin labeling study

**DOI:** 10.1186/s40101-023-00336-1

**Published:** 2023-09-06

**Authors:** Yuki Motomura, Ayaka Fukuzaki, Sanami Eto, Naoki Hirabayashi, Motoharu Gondo, Satoshi Izuno, Osamu Togao, Koji Yamashita, Kazufumi Kikuchi, Nobuyuki Sudo, Kazufumi Yoshihara

**Affiliations:** 1 Department of Human Life Design, Faculty of Design, Kyushu University, 4-9-1 Shiobaru, Minamiku, Fukuoka, 815-8540 Japan; 2 Department of Kansei Science, Graduate School of Integrated Frontier Science, Kyushu University, 4-9-1 Shiobaru, Minamiku, Fukuoka, 815-8540 Japan; 3 Department of Psychosomatic Medicine, Graduate School of Medical Sciences, Kyushu University, Fukuoka, Japan; 4 Department of Psychosomatic Medicine, Kyushu University Hospital, Fukuoka, Japan; 5 Division of Cerebral Integration, Department of System Neuroscience, National Institute for Physiological Sciences, Okazaki, Japan; 6 Department of Clinical Radiology, Graduate School of Medical Sciences, Kyushu University, Fukuoka, Japan

**Keywords:** Alexithymia, Arterial spin labeling, Anterior cingulate cortex, Insular cortex, Salience network, fMRI

## Abstract

**Background:**

Alexithymia, a personality trait characterized by difficulties in identifying and expressing their emotions despite having a range of emotional experiences, can impact individuals’ stress coping mechanisms. While many studies have investigated brain functions associated with specific tasks in relation to emotion processing, research focusing on resting-state brain functions has been limited. Thus, the aim of this study was to investigate the relationship between alexithymia and brain function by analyzing arterial spin labeling (ASL) data obtained during the resting state.

**Methods:**

A brain structural and functional imaging study was conducted on 42 healthy adult men and women using ASL and the 20-item Toronto Alexithymia Scale (TAS-20) questionnaire survey. Cerebral blood flow and functional connectivity values were calculated for regions of interest in the default mode network, saliency network, and central executive network from the ASL data. Correlation analysis was performed with TAS20 scores, and partial correlation analysis was conducted to control for anxiety and depression.

**Results:**

The functional connectivity analysis revealed a negative correlation between the functional connectivity of the right insular cortex and left anterior cingulate cortex and the total score of TAS, as well as difficulty identifying feelings and difficulty describing feeling subscores, indicating that the higher the scores, the weaker the functional connectivity between these regions (T = -3.830, p = 0.0013, R = -0.5180). This correlation remained significant even after controlling for anxiety and depression using partial correlation analysis.

**Conclusion:**

The present study revealed differences in the activity of the Saliency Network at rest as measured by ASL, which were independent of anxiety and depression, and varied depending on the severity of alexithymia. This functional change may underlie the neural basis of decreased emotional processing observed in alexithymia. These findings may contribute to the elucidation of the neural mechanisms of alexithymia, which can lead to social impairments, and suggest the usefulness of ASL measurement as a biomarker of alexithymia.

## Background

Alexithymia is a personality trait characterized by individuals having emotions such as joy, anger, and sadness, but having difficulty recognizing and expressing these emotions. It is considered as one of the personality traits related to emotional processing that has individual differences and is present in the general population [1]. Using the cutoff value of the Toronto Alexithymia Scale, a questionnaire for alexithymia, it has been found that individuals with a high tendency for alexithymia exist to some extent in the general population, at approximately 10.3% [2]. Recent studies have reported on the relationship between problems in interpersonal relationships, empathy deficiency, and healthy individuals. For example, people with a high tendency for alexithymia have weak affection and connection to others, tend to take the initiative in relationships with others, and have difficulty dealing with social problems [3]. It has also been reported that people with a high tendency for alexithymia evaluate pain significantly lower in response to tasks that present others' pain images and have low scores on scales evaluating “Seeing from another person’s perspective” and “empathic concern” in questionnaires measuring capacity for empathy [4]. Such obstacles related to interpersonal relationships and empathy are thought to have negative effects on daily life.

Conversely, alexithymia is deeply related to mental disorders, and previous studies have shown that approximately 45% of people with major depressive disorder (MDD) has alexithymia [5], suggesting that impaired emotional processing is also related to the onset of physical and mental symptoms [6]. It is known that patients with mental and physical disorders accompanied by alexithymia are resistant to psychotherapy [7].

In recent years, brain function studies based on cognitive neuroscience have been attracting attention for elucidating the neural mechanisms of alexithymia [8, 9]. Studies using mainly functional magnetic resonance imaging (fMRI) have been conducted. In a study by Berthoz et al., a decrease in activity was observed in the left anterior cingulate gyrus and prefrontal cortex in response to negative emotional pictures presented using the International Affective Picture System in the alexithymia group [10]. In a study that presented facial expressions of sadness unconsciously, individuals with high alexithymia tendencies showed decreased activity in several brain regions, including the left amygdala, insula, and superior temporal gyrus [11]. Additionally, differences in the activity of the anterior cingulate cortex were observed in response to different types of visual emotional stimuli and emotional valence between individuals with and without alexithymia [12].

This study focuses on the activity of the resting state network (RSN) in individuals with alexithymia. RSNs have been shown to exist in various functions, and each RSN autonomously processes information even during rest and in the unconscious state. Due to the ease of measurement, investigating RSNs as a biomarker has been conducted in diseases such as MDD [13] and neurotic overeating [14]. In a previous study, the functional connectivity between 21 emotion-related brain regions was evaluated as regions of interest (ROI) in resting-state fMRI studies of alexithymia. It was found that alexithymia patients had weak functional connectivity in the left amygdala basal nucleus and the left superior temporal gyrus, as well as in the functional connectivity between the left amygdala basal nucleus and the supplementary motor area (SMA) compared to non-alexithymic patients [15]. Furthermore, a study that examined the functional and structural connectivity between brain regions, with intergroup differences in the amplitude of low-frequency fluctuation (an index that evaluates the amplitude of low-frequency oscillations thought to reflect neural activity in the blood-oxygen-level-dependent [BOLD] signal), found differences in functional connectivity in regions such as the right inferior temporal gyrus to the middle occipital gyrus, left inferior temporal gyrus to the insula, and both anterior cingulate cortices to the frontal pole in the alexithymia group [16].

Thus, given the correlation between alexithymia and mental illness, as well as the lack of empathy, it is considered useful to investigate resting-state brain function for understanding the neural mechanisms underlying this disorder. However, there have been few studies on this topic. Therefore, this study utilized arterial spin labeling (ASL), an imaging technique that uses magnetic resonance imaging (MRI) to obtain perfusion images, to investigate resting-state brain activity. ASL can non-invasively quantify cerebral blood flow (CBF), which is believed to reflect local neural activity and is related to local oxygen consumption and glucose metabolism. ASL is less susceptible to low-frequency noise than functional MRI and can quantitatively measure resting-state brain activity. CBF is a typical parameter obtained from perfusion images, representing blood flow in tissue. There have been few studies using ASL to investigate the RSN in alexithymia. Therefore, this study aims to examine the relationship between alexithymia and brain function by analyzing ASL data obtained during resting-state scans. Specifically, this study investigated the relationship between the alexithymia questionnaire score and the mean CBF value, as well as the functional connectivity between each RSN.

## Methods

### Analysis data

The data analyzed in this study were obtained from an experiment conducted to investigate the integrated effects of nutritional status on the mind and body of patients with eating disorders. Only the data from participants measured as healthy controls for the eating disorder patient group were used in the analysis, while data from the actual patient group were excluded.

### Participants

The participants in the ASL experiment were 49 healthy men and women aged 20 to 56 years (31.75 ± 10.17 years) (16 men and 33 women). Participants were selected for the study based on the following criteria: 1. They were healthy right-handed men and women aged 15 to 69 years; 2. their body mass index (body mass index = weight [kg]/height [m]^2^) was between 18.5 and 25; 3. they had no diagnosis or suspicion of mental or physical illness; 4. they did not have any metal implants such as pacemakers; 5. they did not have any eye diseases including color vision abnormalities; 6. they had no symptoms of claustrophobia; 7. they were not pregnant or had no possibility of being pregnant; 8. the study doctor deemed the participant suitable for the study. Participants who satisfied these conditions participated in this experiment.

### Experimental procedures

The study was conducted from April 2013 to February 2019 at the Department of Psychosomatic Medicine, Kyushu University Hospital, and the Department of Psychosomatic Medicine, Kyushu University Graduate School of Medical Sciences. The research was approved by the research ethics committee at Kyushu University. The participants underwent various tests within 7 days, which included blood biochemistry tests (fasting blood sampling), brain structural imaging tests (MRI, Diffusion Tensor Imaging), brain functional imaging tests (fMRI, Magnetoencephalogram, and ASL), electroencephalography, autonomic nervous system function tests, body composition tests, gastrointestinal transit tests, basal metabolism, and psychological tests. Participants were instructed to keep their head still, not to fall asleep, to keep their eyes closed except when instructed, and to follow other similar instructions during the imaging tests, which took approximately 30 min each. Several questionnaires, such as the 20-item Toronto Alexithymia Scale (TAS-20) and the State-Trait Anxiety Inventory (STAI), were administered for the psychological tests.

### MRI measurement environment

The MRI scans were conducted using a 3.0 T scanner (PHILIPS Achieva, the Netherlands) and the settings for the acquisition of functional (ASL) and structural brain images were as follows:

- ASL image acquisition
  The whole-brain perfusion image was acquired using pseudo-continuous arterial spin labeling. The imaging parameters for the pseudo-continuous arterial spin labeling perfusion imaging were as follows: 2D single-shot gradient-echo echo planar imaging (EPI) in combination with parallel imaging (sensitivity encoding factor 2.0); TR/TE, 4200/8.56 ms; matrix, 64 × 64; FOV, 240 × 240 mm; in-plane resolution, 3.75 × 3.75 mm; 20 slices acquired in ascending order; slice thickness, 6 mm; slice gap, 1 mm; labeling duration, 1650 ms; post-labeling delay (PLD), 2000 ms. Thirty-two pairs of control/label images were acquired and then averaged. The total scan duration for both control and label imaging was 4.72 min.
- Structural image acquisition
  To obtain spatial reference images for analysis, T1-weighted MRI was performed using a T1-weighted magnetization-prepared rapid gradient echo sequence. The imaging parameters were as follows: TR = 7.0 s, TE = 3.2 ms, slice thickness = 1 mm, flip angle = 9°, voxel size = 1 mm × 1 mm × 1 mm, and total scan time = 6.52 min.

### Questionnaires

Below are the details on the questionnaires used in this study.

### TAS-20

TAS-20 is a self-administered questionnaire consisting of 20 items used to evaluate alexithymia. It was originally created by Taylor et al. [17] and is considered to be one of the useful self-report assessment scales worldwide [18].

TAS-20 consists of three subscales: *difficulty identifying feelings* (DIF), d*ifficulty describing feelings* (DDF), and *externally oriented thinking* (EOT). In this study, we used the Japanese version of TAS-20 [18] to conduct the investigation.

### Center for Epidemiologic Studies Depression Scale (CES-D)

CES-D is a self-administered questionnaire consisting of 20 items used to evaluate depressive states. It was developed by the US National Institute of Mental Health to investigate the distribution and related factors of depressive symptoms in the general population. In this study, the Japanese version of CES-D [19] was used for the survey.

### STAI

STAI is a questionnaire consisting of two subscales, STAI-State and STAI-Trait, each with 20 items for a total of 40 items, used to evaluate the degree of anxiety. STAI-State measures state anxiety, which refers to transient anxiety that is experienced in a specific situation and changes rapidly, while STAI-Trait measures trait anxiety, which measures how much anxiety the respondent generally feels and is not affected by situational factors. In this study, the Japanese version of the STAI (Nakazato et al., Published by Sankyobo) was used for the survey.

### ASL analysis

The data of three participants who showed abnormalities in the obtained average CBF map and four participants who did not provide data on TAS-20 were excluded, and the data of 42 participants (age, male-to-female ratio) were used for analysis.

For ASL data analysis, the ASLtbx tool (https://www.cfn.upenn.edu/zewang/ASLtbx.php), SPM12 7771 and its plugin CONN, and Microsoft Excel (for Office 365) were used. Additional analyses were conducted using the statistical software R version 4.1.3 and Microsoft Excel (for Office 365).

### Preprocessing of ASL data and creation of CBF maps

Realignment correction was applied to compensate for head motion during the measurement, and the coregister process was used to match the structural image of the participant with the average image obtained by the realignment correction. After performing the smoothing process with a 6-mm full width at half maximum Gaussian kernel to reduce noise generated by the entire process, the CBF map was created by measuring the difference between the control and label images. The 32 obtained CBF maps were then averaged (hereafter referred to as the mean CBF map) and normalized to the standard brain of MNI templates. As noise was observed in the obtained mean CBF map, the smoothing process was repeated. The mean CBF map was used to investigate the relationship between alexithymia and CBF, and the CBF map was used for ASL functional connectivity analysis, which will be described later.

### The correlation between alexithymia and CBF

Correlation analysis was performed between the average CBF values and TAS-20 using the average CBF map of each participant. The correlation analysis was performed for each voxel, which is the smallest unit of a 3D brain map, and regions of the brain that showed a significant correlation between alexithymia and CBF were estimated based on the results. The average CBF map was calculated using the absolute value of CBF, as we believed that factors such as head size may affect the analysis results. Therefore, in this study, we selected the analysis of covariance option in the global normalization module and performed the analysis by converting the signal of CBF to the ratio of the whole brain signal. The significance level was set at *p* < 0.05 (family-wise error, peak level correction).

### Functional connectivity analysis using ASL

This study focuses on three RSNs based on the “triple network theory” proposed by Menon: the default mode network (DMN), the saliency network (SN), and the central executive network (CEN). Menon has proposed that the relationship between these three core networks plays an important role in many neurological disorders (e.g., chronic pain and depression) [20]. Using these regions as ROIs, functional connectivity analysis was conducted using CBF maps.

The method for calculating functional connectivity involves computing the correlation coefficient between ASL signals from anatomically distant brain regions, and when a significant correlation is observed, functional connectivity between those regions is recognized. Functional connectivity has two types based on the direction of the connection. Positive functional connectivity represents a state where ASL signals from two distant brain regions show positive correlation, indicating a relationship where both regions are simultaneously active when one region is active. Conversely, negative functional connectivity represents a state where ASL signals from two distant brain regions show negative correlation, indicating a relationship where the activity of one region decreases when the other region is active.

The CBF maps were preprocessed and created during the data preprocessing and CBF map creation (1.6.2) through the realignment, coregister, and segmentation processes. Therefore, in this study, only the normalization and smoothing processes were performed for preprocessing. Subsequently, the denoising process was conducted using Conn. In this study, ROI-to-ROI analysis was performed by determining the ROIs in advance and dividing the brain region into arbitrary regions for analysis.

The ROI-to-ROI analysis is one of the fundamental methods in functional connectivity analysis. It is a hypothesis-driven analysis that evaluates the correlation of brain activities by selecting seed regions, and one of its advantages is that it provides a clear meaning when quantifying functional connectivity. However, as the number of ROIs increases, multiple comparison correction is required for all combinations, making it difficult to find significant results, and caution is needed in selecting ROIs. In this study, the brain regions that constitute each RSN (DMN, SN, and CEN) were selected as ROIs, but to address the issue of multiple comparison correction, the ROIs were limited to the core regions of each RSN (DMN, SN, and CEN). The selected regions are shown in Table 1.

**Table 1:** ROIs used for functional connectivity analysis

Network	Brain region name	Abbrev
DMN	Medial Prefrontal Cortex	MPFC
	Right Posterior Cingulate Cortex	R.PCC
	Left Posterior Cingulate Cortex	L.PCC
	Right Precuneus	R.PREC
	Left Precuneus	L.PREC
	Right Inferior Parietal Lobule	R.IPL
	Left Inferior Parietal Lobule	L.IPL
SN	Right Insula	R.INS
	Left Insula	L.INS
	Right Anterior Cingulate Cortex	R.ACC
	Left Anterior Cingulate Cortex	L.ACC
CEN	Right Lateral Prefrontal Cortex	R.LPFC
	Left Lateral Prefrontal Cortex	L.LPFC
	Right Posterior Parietal Cortex	R.PPC
	Left Posterior Parietal Cortex	L.PPC

The ROIs were created with WFU pick atlas tool (https://www.nitrc.org/projects/wfu_pickatlas/) in SPM, following the automated anatomical labeling (AAL) template (https://www.gin.cnrs.fr/en/tools/aal/). In cases where regions (Medial Prefrontal Cortex, Dorsal lateral Prefrontal Cortex, and Posterior Parietal Cortex) could not be created using the AAL template, the corresponding areas were selected from the “network” ROI provided by default in CONN and used in the analysis.

This analysis was conducted in two levels. First, individual-level analysis (1st level analysis) was performed, where ROI-to-ROI analysis was conducted within individuals to calculate functional connectivity values between all selected ROIs. For each functional connectivity value, functional connectivity between each region within DMN was defined as functional connectivity within DMN. Similarly, functional connectivity within SN and CEN was also defined, and these were defined as functional connectivity within the network. Additionally, the combination of each region in DMN (7) and SN (4) was defined as functional connectivity between DMN and SN (a total of 28). Functional connectivity between DMN and CEN and between SN and CEN were also defined in the same manner, and these were defined as functional connectivity between networks. After the individual-level analysis, in the group-level analysis (2nd level analysis), a test was conducted to determine whether the functional connectivity values between the target ROIs were significantly greater than “0” as a group.

### Correlation between alexithymia and functional connectivity

A regression analysis was performed using the total scores and subscale scores (DIF, DDF, and EOT) of TAS-20 for each participant. The regression analysis was conducted for functional connectivity of the significant positive or negative functional connections identified in the ASL functional connectivity analysis at the group level (2nd level analysis) among all ROIs. The significance level was set to 5%, and analysis-level false discovery rate (FDR) correction was used for multiple comparisons.

### Additional analysis

#### Investigation of subscales of TAS-20

To investigate which component of alexithymia is strongly associated with the areas of functional connectivity with alexithymia that were found to be significant, we conducted a regression analysis using the scores of each subscale of TAS-20 (DIF, DDF, and EOT) for each participant, in addition to the analysis of the total TAS score. We set the significance level at 5% and used analysis-level FDR correction for multiple comparisons.

### Partial correlation analysis

There have been reports of a relationship between alexithymia and anxiety or depression. For instance, Lee et al. [21] revealed a positive correlation between the total score of TAS-20, DIF, and STAI-S (state anxiety). In a study conducted with Japanese participants, Kojima et al. [22] reported a positive correlation between TAS-20 and a depression index. Additionally, studies investigating functional connectivity at rest in MDD, anxiety disorder, and anxiety traits have reported abnormal functional connectivity in the DMN in both depression and anxiety [23, 24]. Therefore, even if there is a relationship between alexithymia and functional connectivity, the relationship may be a spurious correlation due to confounding factors such as depression or anxiety. In addition, age and gender have been reported to affect TAS-20 scores [25]. Accordingly, this study conducted additional investigations that considered age, dummy variable of gender, anxiety (STAI) and depression (CES-D) indices for the significant relationship between alexithymia and functional connectivity.

For the analysis method, partial correlation coefficients were calculated between the scores of TAS-20 (total score and DIF score) and the values of functional connectivity, while controlling for the scores of age, gender, STAI-state, STAI-trait, and CES-D as covariates. The pcor function in R version 4.1.3 was used to conduct the partial correlation analysis. The values of functional connectivity were extracted from the individual analysis (1st level analysis) of ASL functional connectivity analysis and used for the analysis. The significance level was set at 5%. Because the STAI, CES-D data for one participant was lost due to a technical error, this analysis was performed on 41 persons.

## Results

### Demographic data

Demographic data for the 42 subjects included in the analysis are shown below (mean ± SD, 28 female): age, 31.19 ± 9.46; TAS20, 31.19 ± 9.46; TAS20-DIF, 31.19 ± 9.46; TAS20-DDF, 31.19 ± 9.46; TAS20-EOT, 31.19 ± 9.46; CES-D, 31.19 ± 9.46; STAI-state, 31.19 ± 9.46; STAI-trait, 31.19 ± 9.46. Four participants scored above the TAS-20 cut-off point (61), with a maximum score of 63. No participant scored above 70, confirming that alexithymia tendencies were generally within the healthy range in the current group of participants.

### Correlation between alexithymia and CBF

The analysis of the correlation between alexithymia and CBF did not reveal any significant voxel with a significant relationship.

### ASL functional connectivity analysis

**Fig. 1:**
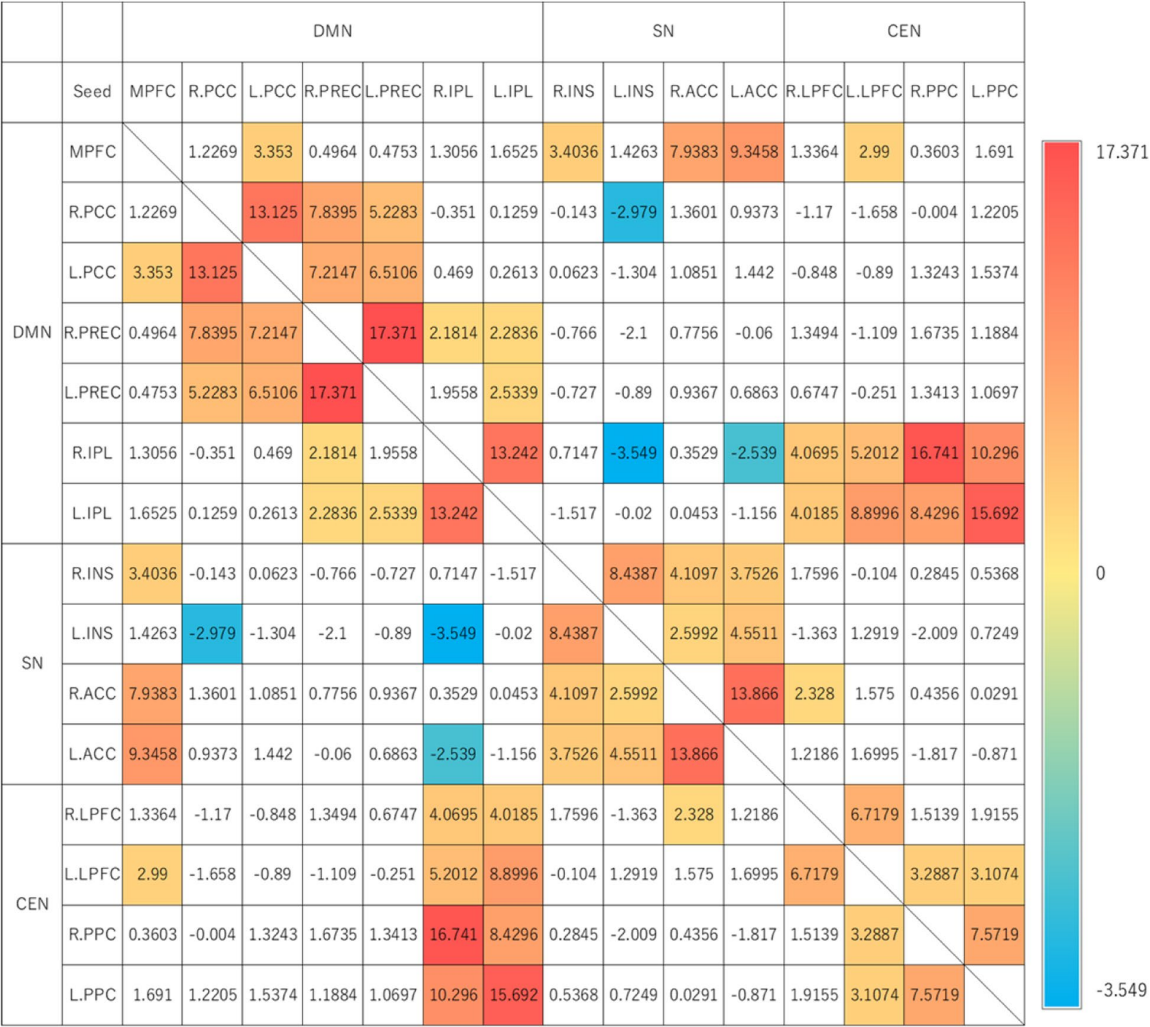
Results of ASL functional connectivity analysis (group-level analysis). The results of the group analysis (2nd level analysis) are shown in the table, where the presence or absence of functional connectivity between regions is indicated by color (*p* < 0.05 analysis-level FDR correction). The numbers in the table represent t-values. The red colors indicate significant positive functional connectivity, the blue colors indicate significant negative functional connectivity, and the color intensity reflects the strength of the functional connectivity. The uncolored areas indicate no significant functional connectivity. See Table 1 for abbreviations of ROIs. TAS-20 = The 20-item Toronto Alexithymia Scale; DMN = default mode network; SN = salience network; CEN = central executive network; FDR = false discovery rate; ROI = region of interest

Figure 1 shows the results of functional connectivity analysis in ASL data. ROI-to-ROI analysis was conducted using core regions that comprise the DMN, SN, and CEN as ROIs. Positive functional connectivity was observed in many regions within networks, and negative functional connectivity was observed between the DMN and SN in some cases.

### Correlation between alexithymia and functional connectivity

**Fig. 2:**
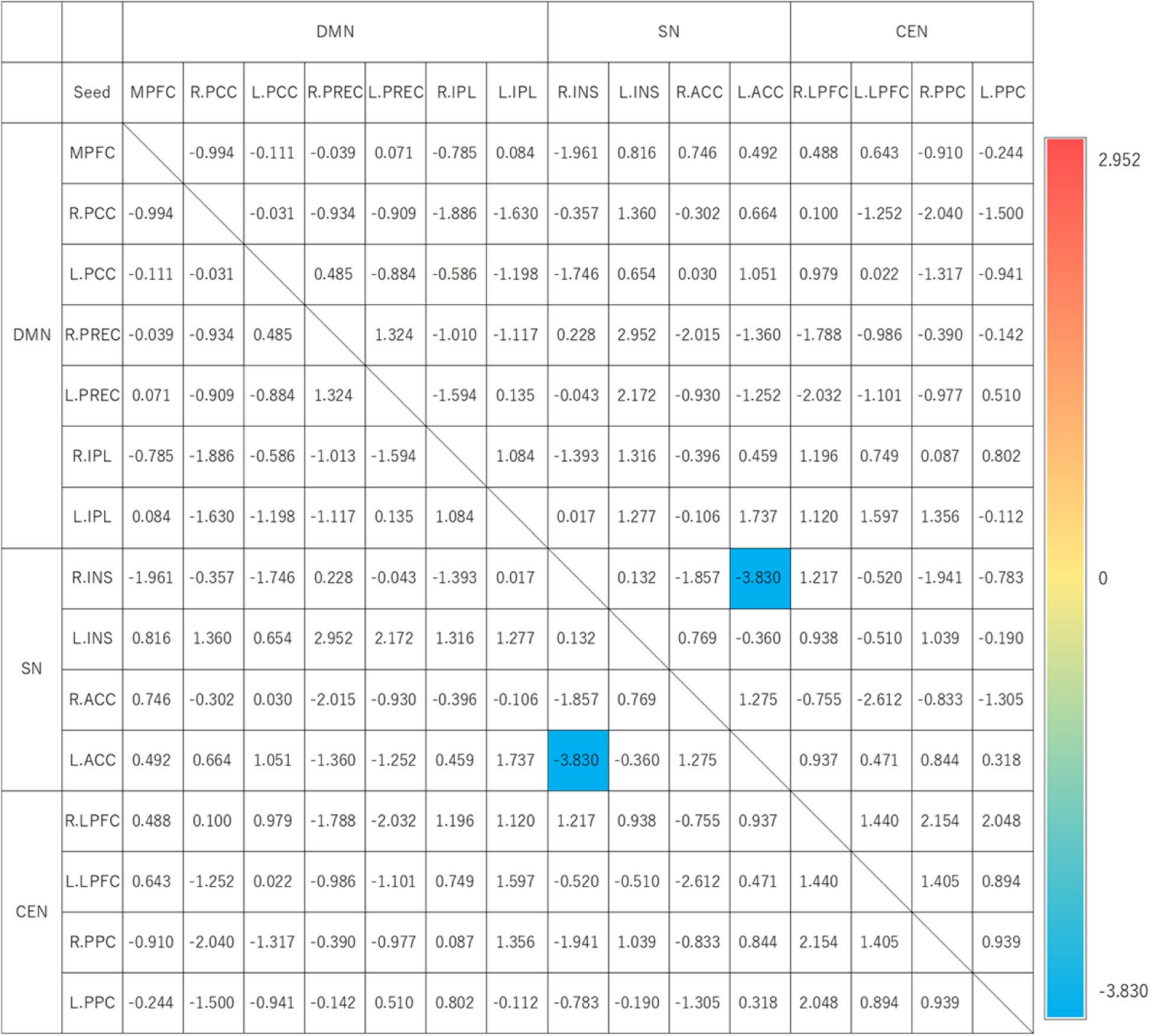
Results of the regression analysis on the relationship between alexithymia and functional connectivity. The table shows the results of the regression analysis between the TAS-20 total score and functional connectivity. The numbers in the table represent t-values. The color indicates the nature of the correlation, with red indicating a significant positive correlation, blue indicating a significant negative correlation, and the intensity of the color representing the strength of the correlation. Blank cells indicate areas where no significant results were found in the analysis (*p* < 0.05 analysis-level FDR correction). See Table 1 for abbreviations of ROIs. TAS-20 = The 20-item Toronto Alexithymia Scale; DMN = default mode network; SN = salience network; CEN = central executive network; FDR = false discovery rate; ROI = region of interest

The results of the regression analysis concerning the correlation between alexithymia and functional connectivity are shown in Fig. 2.

**Fig. 3:**
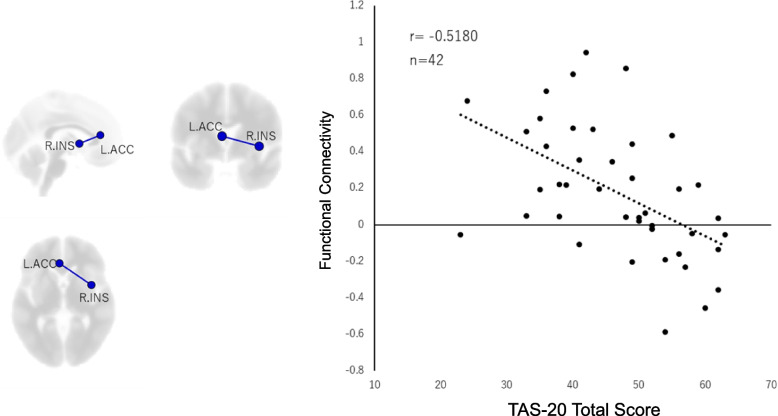
Correlation between alexithymia and functional connectivity between the right insula and the left anterior cingulate cortex. The left side shows the location of the ROIs on a 3D brain map, while the right side shows the scatter plot. The total score of TAS-20 exhibited a significant negative correlation with the functional connectivity between the right insula and the left anterior cingulate cortex.(r[40] = 0.52, *p* = 0.0013, FDR corrected) FDR = false discovery rate; ROI = region of interest; R.INS = right Insula; L.ACC = left anterior cingulate cortex

Based on the results in Fig. 2, a scatter plot was created to show the significant functional connections between brain regions identified by functional connectivity analysis and the total score of TAS-20 with a significant positive or negative correlation (Fig. 3).

The scatter plot revealed a significant negative correlation (T = -3.830, p-FDR = 0.0013, r = -0.5180) between the total score of TAS-20 and the functional connectivity between the right insula and the left anterior cingulate cortex.

### TAS-20 sub-scale analysis

The DIF and DDF scores of TAS-20 were negatively correlated with the functional connectivity between the right insula and the left anterior cingulate cortex (T = -4.004, p-FDR = 0.0008, r = -0.5349; T = -3.142, p-FDR = 0.0094, r = -0.4450). Furthermore, the DDF score showed a negative correlation with the functional connectivity between the right insula and the right anterior cingulate cortex (T = -2.885, p-FDR = 0.0094, r = -0.4150). No significant correlation was found between EOT score and functional connectivity.

**Fig. 4:**
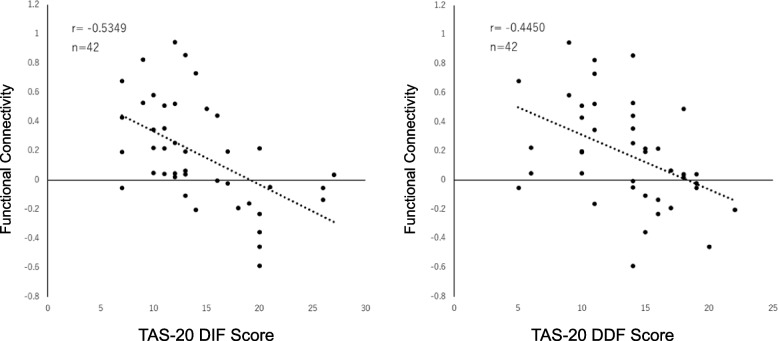
Correlation between subscales of alexithymia and functional connectivity between the right insular cortex and left anterior cingulate cortex. On the left, a scatter plot shows the correlation between TAS-20 DIF score and the functional connectivity between the right insula and the left anterior cingulate cortex. On the right, a scatter plot shows the correlation between TAS-20 DDF score and the functional connectivity between the same regions. Both DIF and DDF scores showed a significant negative correlation with functional connectivity. (DIF: r[40] = 0.53, *p* = 0.0008, FDR corrected; DDF: r[40] = 0.45, *p* = 0.0094, FDR corrected) TAS-20 = The 20-item Toronto Alexithymia Scale; DIF = Difficulty Identifying Feeling subscale; DDF = Difficulty Describing Feelings subscale; FDR = false discovery rate; ROI = region of interest; R.INS = right insula; L.ACC = left anterior cingulate cortex

**Fig. 5:**
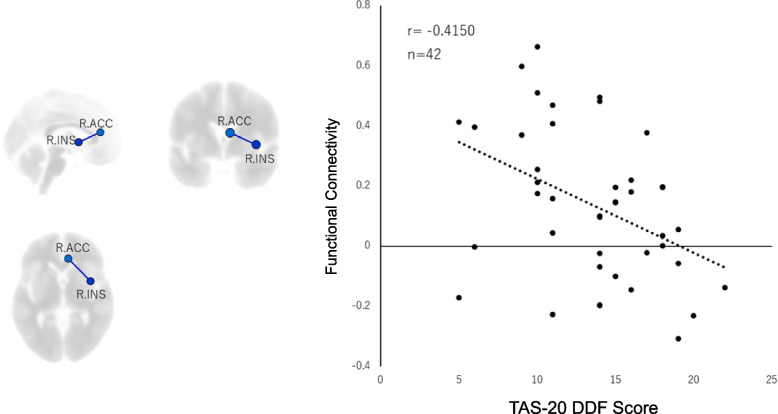
Correlation between subscales of alexithymia and functional connectivity between the right insular cortex and right anterior cingulate cortex. The left shows the location of the region of interest (ROI) on a 3D brain map, and the right panel shows a scatter plot. A significant negative correlation was observed between the DDF score of TAS-20 and the functional connectivity between the right insula and the right anterior cingulate cortex. (r[40] = 0.42, *p* = 0.0094, FDR corrected). TAS-20 = The 20-item Toronto Alexithymia Scale; DDF = Difficulty Describing Feelings subscale; FDR = false discovery rate; ROI = region of interest; R.INS = right insula; R.ACC = right anterior cingulate cortex

Scatter plots were created for the results that showed a significant negative correlation (Figs. 4 and 5).

### Partial correlation analysis

Table 2 shows the correlation coefficients before controlling for age, gender, anxiety and depression, while Table 3 shows the partial correlation coefficients when age, gender, STAI-state, STAI-trait, and CES-D scores were used as control variables in the partial correlation analysis. After controlling for age, gender, anxiety and depression, a significant negative correlation was found between the total score of TAS-20 and the functional connectivity between the right insula and left anterior cingulate cortex (T = -2.2930, *p* = 0.02858, r = -0.3660). Similarly, a significant negative correlation was found between the DIF score and the functional connectivity between the right insula and left anterior cingulate cortex (T = -2.624, *p* = 0.01293, r = -0.4103). A significant negative correlation was also found between the DDF score and the functional connectivity between the right insula and right anterior cingulate cortex (T = -2.2412, *p* = 0.03165, r = -0.3588), and a marginally significant correlation was found between the DDF score and the functional connectivity between the right insula and left anterior cingulate cortex (T = -1.7812, *p* = 0.08380, r = -0.2922).

**Table 2:** Relationship between alexithymia and functional connectivity before controlling for age, gender, anxiety and depression

Functional Connectivity	TAS-20 score		
	Total Score	DIF	DDF
Right Insula-left anterior cingulate cortex	-0.5180^**^	-0.5349^***^	-0.4450^**^
Right insula-right anterior cingulate cortex			-0.4150^**^

1)*n* = 42

2)^*^p < 0.05, ^**^p < 0.01, ^***^p < 0.001

**Table 3:** Relationship between alexithymia and functional connectivity after controlling for age, gender, anxiety and depression

Functional Connectivity	TAS-20 score		
	Total score	DIF	DDF
Right Insula-left anterior cingulate cortex	-0.3660^*^	-0.4103^*^	-0.2922^†^
Right insula-right anterior cingulate cortex			-0.3588^*^

1)*n* = 41

2)^†^ *p* < 0.1, ^*^ *p* < 0.05

## Discussion

This study investigated the association between alexithymia and brain function by analyzing ASL data obtained during resting state. Although no significant correlation was found between CBF measured by ASL and alexithymia, a negative correlation was found between the total score and scores on the DIF subscale (difficulty identifying feelings) of the TAS-20 and the functional connectivity between the right insula and left anterior cingulate cortex. Additionally, a negative correlation was found between the DDF subscale score (difficulty describing feelings to others) of the TAS-20 and the functional connectivity between the right insula and right anterior cingulate cortex.

The insula and anterior cingulate cortex are regions that constitute the SN, and it has been found that they are often co-activated in various situations, including interoception, self-recognition, and emotion recognition [26]. Terasawa et al. [27] examined brain activity during the evaluation of sentences related to emotional states and bodily conditions (e.g., “I am happy,” “My pulse is fast”) and found that the right anterior insula and bilateral anterior cingulate cortex were activated in the evaluation of the current emotional state. Furthermore, SN is now recognized as part of the emotional network [28]. Previous fMRI studies using emotional stimuli have reported that the activity of the anterior cingulate cortex and insula is related to alexithymia [10, 11, 29]. Therefore, based on the fact that the functional connectivity between the insula and anterior cingulate cortex was weakened as the scores of DIF (difficulty identifying feelings) and DDF (difficulty describing feelings) in the present study increased, it can be considered that the relationship between alexithymia and functional connectivity observed in this study is closely related to emotion processing.

The negative correlation between functional connectivity in the insula-anterior cingulate cortex and alexithymia was observed, and the insula functions in change perception in internal bodily states and promoting awareness [30]. In contrast, the anterior cingulate cortex has been reported to be involved in the evaluation, regulation, and cognitive control of emotions [31, 32]. Based on these findings, it can be suggested that individuals with a high tendency towards alexithymia may have problems with the coordination between the insula, which is involved in the perception of emotions, and the anterior cingulate cortex, which is involved in emotional control.

Based on the results of this study, the anterior cingulate cortex was associated with alexithymia on both the left and right sides, while the insula was only associated with alexithymia on the right side. Several previous studies have reported that the right insula in particular is involved in the recognition of emotional states [26, 33, 34]. If the observed association between alexithymia and functional connectivity is closely related to emotions, it is possible that the right insula plays an important role in emotion recognition even during a resting-state brain activity.

Prior studies using fMRI data have not demonstrated a relationship between alexithymia and SN; however, a comparison of functional connectivity between resting-state fMRI and ASL data has revealed some differences in the same resting-state brain function data. Specifically, fMRI showed strong functional connectivity in the posterior regions of the default mode network (precuneus and bilateral angular gyrus), whereas ASL showed strong functional connectivity in the medial prefrontal cortex [35], suggesting that different regions are sensitive to each method. This difference in functional connectivity between fMRI and ASL has also been confirmed in studies using different analysis methods, such as seed-to-voxel analysis, and it is believed to be related to differences in MRI imaging protocols [36]. In this study, ASL showed a relationship between alexithymia and the frontal brain regions, such as the anterior cingulate cortex and insula. Furthermore, ASL has lower signal-to-noise ratio compared to fMRI [37], and ASL has less data compared to fMRI, which may have contributed to the differences in the results. The difference in data may be due to the fact that ASL needs to acquire two types of images, control and label images, to calculate the CBF map. Therefore, the differences in the characteristics of the measurement methods between fMRI and ASL may have influenced the results of this study.

There are several limitations to this study. The first limitation concerns the selection of ROIs for functional connectivity analysis. When ROIs are subdivided into smaller regions, the results may differ. In this study, ROIs were selected based on the AAL atlas, without considering the subregions of each area. For example, the anterior cingulate cortex can be subdivided into multiple regions that belong to different networks [38]. Specifically, the ventral anterior cingulate cortex is part of the default mode network (DMN) [39], while the dorsal anterior cingulate cortex is identified as part of the SN [40]. The posterior cingulate cortex can also be subdivided into several regions, with the ventral subregion showing strong functional connectivity with the rest of the DMN, and the dorsal subregion showing high functional connectivity with the prefrontal network involved in cognitive control [41]. Therefore, as functional roles may differ within a single area, future studies examining functional connectivity within and between networks should also consider the subregions of each area.

The second limitation of this study pertains to the need for caution when measuring alexithymia. In this study, the degree of alexithymia was assessed using the TAS-20 questionnaire. While the TAS-20 is a highly useful evaluation scale worldwide [18], studies have emphasized the limitations of measuring alexithymia through a questionnaire survey. Specifically, a study that measured alexithymia using both structured interviews and questionnaire surveys found that although the total scores were correlated, there were discrepancies in the results between the clinical assessment of emotional recognition and the DIF score (difficulty in identifying feelings) of the TAS-20 [42]. As both the fMRI and ASL analyses in this study showed a correlation between the DIF score and functional connectivity, the accuracy of evaluating the degree of difficulty in identifying emotions may have influenced the analysis results. Thus, in the future, more careful consideration is necessary when measuring alexithymia.

The third limitation is that no assessment of sleepiness was performed. Since the main aim of this entire project was to compare patients with eating disorders, the evaluation of sleepiness was not included in the protocol. Sleepiness has been shown to affect functional connectivity [43]. Although this study is a comparison in the absence of sleep-related interventions and therefore is not expected to have the same impact as previous studies, it is possible that sleepiness at the time of imaging may have had a small impact.

One of the indicators of brain activity used in this study, functional connectivity, is estimated based on the correlation between parameters of neural activity such as ASL signals, and it is difficult to determine whether the functional connectivity between two regions is a direct or causative relationship [44]. In the future, investigating other approaches, such as effective connectivity, which can estimate direct causality between functionally connected regions, will be useful for elucidating the neural mechanisms of alexithymia. Furthermore, given the need for careful consideration in determining whether changes in functional connectivity are disease-specific or dependent on the state of arousal [45], it is important to conduct further research in this field to provide more robust evidence.

## Conclusions

We have demonstrated that there are differences in the activity of the SN during rest, as measured by ASL, that are independent of anxiety and depression and are based on the degree of alexithymia. This functional change may underlie the neural basis of reduced emotion processing observed in alexithymia. These results have the potential to contribute to the elucidation of the neural mechanisms of alexithymia, which can lead to social impairment, and demonstrate the usefulness of ASL measurements as a biomarker for alexithymia; in particular, based on previous studies showing that impaired emotional processing is associated with the development of psychosomatic and mental disorders [6], and that patients with psychosomatic and mental disorders with apraxia are resistant to psychotherapy [7], ASL imaging as a biomarker may contribute to treatment selection and differentiation of high-risk groups.

## Data Availability

The datasets generated during and/or analyzed during the current study are available from the corresponding author on reasonable request.

## Abbreviations

AAL: Automated anatomical labeling
ASL: Arterial spin labeling
BOLD: Blood-oxygen-level-dependent
CBF: Cerebral blood flow
CEN: Central executive network
CES-D: Center for Epidemiologic Studies Depression Scale
DDF: Difficulty describing feelings
DIF: Difficulty identifying feelings
DMN: Default mode network
EOT: Externally oriented thinking
ET: Echo time
FDR: False discovery rate
fMRI: Functional magnetic resonance imaging
MDD: Major depressive disorder
MRI: Magnetic resonance imaging
ROI: Regions of interest
RSN: Resting state network
SMA: Supplementary motor area
SN: Saliency network
STAI: State-Trait Anxiety Inventory
TAS-20: 20-Item Toronto Alexithymia Scale
TR: Repetition time
